# Effects of a Sexual Health Literacy Intervention on Preventive Sexual Risk Behaviors Among Lower Secondary School Students: A Quasi-Experimental Study

**DOI:** 10.3390/bs16060873

**Published:** 2026-06-01

**Authors:** Piponticha Huangmit, Nannapat Ketkosan, Chakkrit Ponrachom

**Affiliations:** Faculty of Education, Kasetsart University, Bangkok 10900, Thailand; piponticha.hu@ku.th (P.H.); nannapat.k@ku.th (N.K.)

**Keywords:** health literacy program, adolescents, preventive behaviors, Thailand

## Abstract

Preventing sexual risk behaviors among early adolescents is essential, as it may delay premature sexual initiation and reduce the incidence of sexually transmitted infections. This quasi-experimental study aimed to evaluate the effectiveness of a sexual health literacy promotion program in enhancing preventive sexual risk behaviors among lower secondary school students. The sample consisted of 114 eighth-grade students selected through multistage sampling and assigned to either an intervention group or a control group, with 57 participants in each group. The intervention group received the sexual health literacy promotion program (CoMMIT program), based on the health literacy framework and comprising five structured learning activities. The control group received the standard curriculum. Data were collected using demographic, sexual health literacy, and sexual risk behavior prevention questionnaires. Measurements were conducted at pre-intervention, post-intervention, and one-month follow-up. Data were analyzed using two-way mixed-design repeated measures ANOVA and independent *t*-tests. The intervention group demonstrated significantly higher mean scores for sexual health literacy and preventive sexual risk behaviors at post-intervention and one-month follow-up compared with baseline and the control group (*p* < 0.001). Large effect sizes were observed for sexual health literacy (η^2^ = 0.932) and sexual risk behaviors prevention (η^2^ = 0.791). The CoMMIT program effectively improved sexual health literacy and preventive sexual risk behaviors among lower secondary school students and is recommended for implementation in secondary schools to promote adolescent sexual health.

## 1. Introduction

Adolescence is a period characterized by extensive development across multiple domains and represents a critical stage for establishing a foundation for good health. Adolescents experience rapid physical, psychological, social, and psychosocial development (Demaria et al., 2024). Although adolescence is recognized as a period of major transition, adolescents continue to face a high risk of mortality and morbidity (World Health Organization, 2024a). Contemporary adolescents commonly engage in health-risk behaviors, including unhealthy dietary consumption (Soliman et al., 2022), substance use (Nawi et al., 2021), and sexual risk behaviors (World Health Organization, 2024b). In particular, sexual risk behaviors are influenced by multiple life factors, including pubertal hormonal changes that stimulate sexual desire and increase interest in romantic and sexual relationships (Belošević, 2023), leading adolescents to curiosity, sexual experimentation, and eventually engagement in sexual intercourse (Frankenbach et al., 2022). These behaviors have received global attention because of their long-term negative consequences (Moilanen et al., 2010). The World Health Organization (WHO) has reported more than one million cases of sexually transmitted diseases (STDs) per day worldwide (World Health Organization, 2025). In addition, unintended pregnancy, unsafe abortion, sexual violence (Myat et al., 2024), and adverse sexual and reproductive health outcomes remain prevalent societal problems (Kelecha et al., 2024). Consequently, adolescence constitutes a life stage associated with multiple adverse health outcomes that affect not only adolescents themselves but also their families, communities, and broader socioeconomic systems (United Nations Children’s Fund, 2021; Marino et al., 2022).

At present, numerous international and national organizations have implemented programs aimed at preventing adolescent sexual risk behaviors, particularly unsafe sexual practices and substance use (Centers for Disease Control and Prevention, 2024). However, despite these efforts, sexual risk behaviors remain prevalent, suggesting that interventions relying primarily on information provision or externally driven behavioral control may be insufficient to achieve sustainable behavior change. Previous interventions have largely emphasized sexual knowledge transmission, abstinence promotion, or risk-avoidance approaches, whereas relatively limited attention has been given to strengthening adolescents’ competencies in accessing, critically evaluating, communicating, and applying sexual health information in real-life situations. Although some interventions have demonstrated improvements in sexual knowledge and attitudes, evidence regarding comprehensive sexual health literacy interventions remains limited, particularly among younger adolescents in school settings. This limitation has increased recognition of the importance of individual-level determinants that shape adolescents’ capacity to make informed and responsible health decisions. Evidence indicates that such determinants play an important role in promoting protective sexual behaviors among adolescents (El Kazdouh et al., 2019). Because social and environmental contexts continuously evolve (World Health Organization, 2023), identifying and strengthening key individual-level determinants is essential for developing sustainable and effective interventions for sexual risk reduction (Kabiru et al., 2024). Among these determinants, health literacy has been widely recognized as a key factor influencing health behavior and health outcomes.

Health literacy refers to the cognitive and social skills that enable individuals to access, understand, appraise, and apply health information to make appropriate health decisions and maintain good health (Liu et al., 2020; Smith et al., 2025). As a multidimensional construct, health literacy extends beyond the acquisition of knowledge and includes critical thinking, problem-solving, and decision-making competencies required for health management. Within this framework, sexual health literacy represents a domain-specific extension of general health literacy focused on sexual and reproductive health. General health literacy refers to overall abilities to manage health information across multiple health domains, whereas sexual health literacy specifically refers to competencies required to navigate sexual health-related information, contexts, and decisions. Importantly, sexual health literacy differs from sexual health knowledge. Sexual health knowledge primarily refers to factual information regarding sexual health, whereas sexual health literacy involves higher-order abilities to access, critically evaluate, interpret, and apply information in real-life situations. These abilities include communication, self-management, media literacy, and informed decision-making regarding sexual behavior. Therefore, adolescents with high sexual health literacy are not only informed but also able to translate knowledge into appropriate and context-sensitive behavioral actions. Sexual health literacy encompasses multiple interrelated competencies, including information access, comprehension, communication skills, self-management, media literacy, and decision-making skills (Nutbeam, 2000). Adolescents with adequate sexual health literacy are more likely to engage in protective sexual behaviors, such as avoiding substance and alcohol use, avoiding sexually explicit or stimulating media, and avoiding high-risk intimate situations (Buja et al., 2020). This suggests that sexual health literacy functions not merely as knowledge acquisition but as an applied capacity that supports behavioral regulation in complex social environments.

The health literacy framework was selected to guide this study because it provides a more comprehensive explanation of health behavior than traditional behavioral theories. While models such as the Theory of Planned Behavior and Social Cognitive Theory emphasize attitudes, intentions, and observational learning, they do not fully capture individuals’ abilities to access, interpret, and critically apply health information in real-world decision-making contexts. In contrast, the health literacy framework integrates cognitive, behavioral, and social dimensions of health competence, making it particularly suitable for understanding and influencing complex behaviors such as adolescent sexual decision-making.

Most studies examining sexual health literacy interventions have been conducted in Western or high-income countries, limiting the generalizability of findings to sociocultural contexts in Southeast Asia. Cultural norms, family communication patterns, educational systems, and access to sexual health information may substantially influence adolescents’ sexual health literacy and sexual decision-making behaviors. In Thailand, adolescent sexual and reproductive health remains an important public health concern. Previous studies among Thai adolescents have reported persistent sexual risk behaviors, including inconsistent contraceptive use, multiple sexual partnerships, and exposure to sexual media among school-aged adolescents (Thepthien & Celyn, 2022; Srijaiwong et al., 2017). Studies conducted in Thailand have also highlighted the important role of sexual health literacy and media literacy in influencing adolescents’ sexual behaviors and decision-making processes (Narkbubpha et al., 2025). However, existing sexuality education programs in Thailand continue to focus primarily on biological knowledge and risk avoidance, with relatively limited emphasis on strengthening communication, critical appraisal, self-management, and decision-making competencies related to sexual health. Furthermore, empirical evidence regarding structured sexual health literacy promotion interventions among Thai lower secondary school students remains limited. Early adolescence represents a critical developmental stage during which attitudes, behavioral patterns, and decision-making competencies related to sexuality begin to emerge. Therefore, interventions delivered during lower secondary school may provide important opportunities to establish protective sexual health behaviors before engagement in higher-risk activities occurs. Schools represent a key setting for promoting sexual health literacy because they provide equitable and structured opportunities for adolescents to develop essential health competencies in a safe and supportive environment. Integrating sexual health literacy into school-based self-care promotion programs may help lower secondary school students access accurate sexual health information, strengthen critical thinking and decision-making skills (Smith et al., 2025), reduce sexual risk behaviors such as unprotected sexual intercourse and unintended pregnancy, and enhance communication and refusal skills.

Unlike conventional sexuality education programs that primarily emphasize factual knowledge and risk avoidance, the CoMMIT program was designed to strengthen multidimensional sexual health literacy competencies, including communication, critical appraisal, self-management, media literacy, and decision-making skills through interactive and participatory learning activities. Therefore, culturally relevant and evidence-based interventions grounded in the health literacy framework are needed to strengthen adolescents’ capacity for sexual risk prevention in the Thai context. The findings of this study may contribute to the growing evidence base regarding sexual health literacy interventions and inform the development of school-based adolescent sexual health promotion strategies in Thailand and similar sociocultural contexts.

Despite increasing recognition of the importance of sexual health literacy in adolescent sexual risk prevention, existing interventions have largely focused on knowledge transmission and risk avoidance approaches, with limited emphasis on developing multidimensional sexual health literacy competencies such as communication, critical appraisal, self-management, media literacy, and decision-making skills. In addition, most available evidence has been generated in Western or high-income country contexts, limiting its applicability to Southeast Asian settings such as Thailand. Furthermore, empirical studies examining structured school-based sexual health literacy promotion programs among lower secondary school students remain limited. Therefore, there is a need for culturally appropriate, theory-based interventions that strengthen adolescents’ sexual health literacy and support sustainable behavioral change. Therefore, this study aimed to evaluate the effectiveness of a sexual health literacy promotion program in enhancing preventive sexual risk behaviors among lower secondary school students.

## 2. Materials and Methods

### 2.1. Study Design

This study employed a quasi-experimental design using a two-group comparison with three measurements: baseline (pre-intervention), post-intervention, and a 1-month follow-up period. Participants were selected using multistage sampling procedures that combined purposive and random sampling techniques. The intervention group received the sexual health literacy promotion program (CoMMIT program) developed by the researchers, whereas the control group received the school’s routine activities. Eligible participants met the following inclusion criteria: being a Grade 8 student, aged 13–15 years; ability to read, listen, and write in Thai; ownership of and ability to use a smartphone with internet access to participate in research activities; and good general health. Parental informed consent and informed assent from the participating students were obtained prior to participation. No formal exclusion criteria were applied. Participants were considered withdrawn if they voluntarily left the study, were unable to complete the intervention activities, provided incomplete data, or experienced serious illness during the study period.

### 2.2. Sampling

Multistage sampling was used to select the study sample. The six districts of Nonthaburi Province, Thailand, were grouped into three clusters based on similar population sizes. One district with a large adolescent population, Mueang Nonthaburi District, was purposively selected due to its relevance to the study context. Mueang Nonthaburi District, which comprised seven schools, was selected. Second, two extra-large schools were selected using simple random sampling. To minimize contamination between participants, one school was assigned as the intervention group and the other as the control group. This approach was used to reduce the possibility of information exchange and intervention-related interactions between participants across study conditions. However, group assignment to the intervention and control conditions was not randomized, as assignment was conducted at the school level to reduce contamination between participants, consistent with a quasi-experimental design. Third, classrooms within each selected school were chosen using simple random sampling. Students who met the inclusion criteria were invited to participate in the study. Baseline equivalence between the intervention and control groups was assessed prior to the intervention to ensure comparability.

Although group assignment was conducted at the school level, multiple classrooms and individual students were included within each school. Therefore, the primary unit of analysis in this study was the individual student rather than the school. However, because only two schools were included in the study, potential clustering effects at the school level could not be fully accounted for statistically. Multilevel analysis was not feasible with such a limited number of clusters. Therefore, the findings should be interpreted cautiously, and future studies should include a larger number of schools to better address clustering effects and improve the robustness of causal inference. Because participants were recruited from different schools, contextual differences between schools (e.g., school environment, teaching practices, and peer norms) may not have been fully controlled and should be considered a limitation of the study.

**Figure 1:**
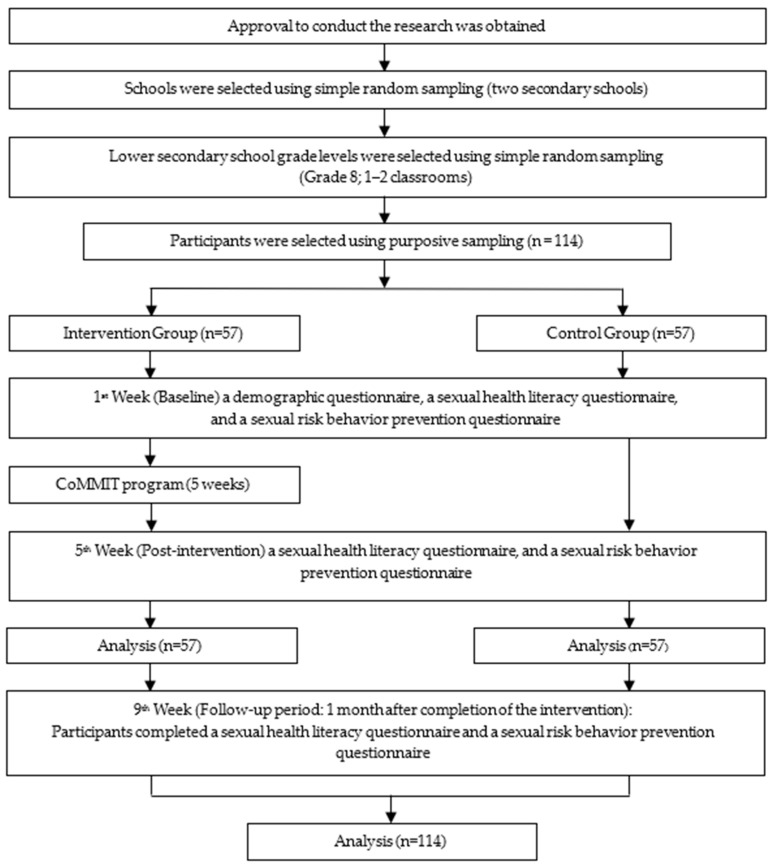
Flowchart of the research process.

The sample size was calculated using the G*Power version 3.1.9.7 program. The parameters were set at a statistical power of 0.80, an effect size of 0.5, and a confidence level of 95% (Cohen, 1992). The selected effect size represented a moderate magnitude based on Cohen’s conventional criteria and was considered appropriate for behavioral research in the absence of prior empirical estimates. The required sample size was 102 participants, divided equally into an intervention group and a control group (51 participants per group). To account for potential attrition, an additional 10% was added, resulting in six additional participants per group. Consequently, the final sample comprised 114 participants, with 57 participants in each group (Figure 1).

Participants in the intervention group received the CoMMIT program as part of integrated learning activities within the student development program, implemented over a 5-week period with one session per week (approximately 100 min per session). All sessions were delivered according to a standardized protocol to ensure consistency across implementation. Attendance was recorded to monitor adherence. No dropouts occurred, and all participants completed all sessions.

Participants in the control group received routine school-based activities within the standard student development program conducted by teachers. These activities were delivered over the same 5-week period, with one session per week (approximately 100 min per session). The routine program included general classroom guidance, school assemblies, extracurricular activities, and moral and civic education, consistent with the usual school curriculum. These activities were not modified for research purposes and did not include any structured sexual health literacy content or components comparable to the CoMMIT intervention. In addition, no specific elements related to sexual health literacy, decision-making skills, or sexual risk behavior prevention were systematically delivered during the study period.

### 2.3. Research Instruments

Data were collected using a self-administered structured questionnaire developed based on a comprehensive review of relevant literature and previous studies. The instrument was refined to ensure contextual appropriateness and consisted of three parts: (a) demographic data, (b) sexual health literacy, and (c) sexual risk behavior prevention.

(a) Demographic data: This section included 8 items assessing individual and environmental characteristics, including gender, age, romantic relationship status, parental marital status, living arrangements, allowance, leisure activities, and internet and social media use.
(b) Sexual Health Literacy: This questionnaire assessed sexual health literacy, comprising six domains: access to information (Access), cognitive understanding (Cognitive), communication skills (Communication Skill), self-management (Self-Management), media literacy (Media Literacy), and decision-making skills (Decision Skill).

This scale consisted of 45 items across six domains: access to information (6 items), cognitive understanding (12 items), communication skills (6 items), self-management (6 items), media literacy (6 items), and decision-making skills (9 items). Items were measured using mixed formats: (1) a 5-point Likert-type scale (“always” to “never”), (2) multiple-choice items scored as correct (1) or incorrect (0), and (3) scenario-based items scored from 1 to 4. Total scores ranged from 33 to 168, with higher scores indicating higher levels of sexual health literacy. Both total and domain-specific scores were calculated. For inferential analysis, total scores were primarily used to examine overall differences between groups, while domain-specific scores were used for descriptive interpretation.

(c) Sexual Risk Behavior Prevention: This scale consisted of 18 items (13 positively worded and 5 negatively worded) covering three domains of desirable preventive behaviors: avoidance of substance use and alcohol consumption, avoidance of sexually stimulating media, and avoidance of intimate physical contact in private settings. Responses were measured using a 5-point frequency scale. Reverse scoring was applied to negatively worded items. Higher scores indicated better preventive behavior.

The sexual health literacy and sexual risk behavior prevention questionnaires (Parts b and c) achieved a content validity index ranging from 0.60 to 1.00, as evaluated by five experts. The instruments were pilot-tested with 40 participants who were representative of the target population but not included in the main study sample. The questionnaires demonstrated acceptable reliability, with Cronbach’s alpha coefficients of 0.882 and 0.784, respectively, indicating that the instruments met the required standards of reliability for research purposes (Ponterotto & Ruckdeschel, 2007).

To minimize measurement bias, the instruments were designed to assess general competencies across multiple domains rather than directly replicate specific intervention activities.

### 2.4. Intervention

To develop the content for the sexual health literacy promotion program, an extensive review of the literature on sexual health literacy was conducted, including key concepts, dimensions of sexual health literacy, critical thinking skills, communication related to the prevention of sexual risk behaviors, and the influence of online media that may lead to risk behaviors. The content was then reviewed and refined to enhance clarity and comprehensibility. The material was summarized and reorganized into accessible formats, including video-based media, narratives, videos, animations, and age-appropriate activities.

The program content was structured into five sequential learning modules (Table 1) and delivered through interactive and participatory learning approaches to promote engagement and practical skill development.

The CoMMIT program derives its name from the English word “CoMMIT,” meaning commitment, promise, and dedication. This concept reflects individuals who possess sexual health literacy, characterized by readiness to manage various situations through self-commitment and personal determination. Accordingly, the CoMMIT program was designed as a set of learning activities that foster personal commitment and self-promise to become a responsible individual—in this context, an individual with sexual health literacy—thereby facilitating effective modification of preventive sexual risk behaviors in the future.

### 2.5. Data Analysis

Data were screened for completeness and analyzed using a significance level of α = 0.05. Descriptive statistics (frequency, percentage, mean, SD) summarized participant characteristics. Inferential analyses included independent *t*-tests for between-group comparisons and two-way mixed-design repeated measures ANOVA for within- and between-group effects over time. Assumptions of normality and homogeneity of variance were tested prior to analysis. Given the clustered sampling structure (students nested within schools), and the limited number of clusters (two schools), multilevel modeling was not feasible. Therefore, standard ANOVA was applied at the individual level, and findings should be interpreted with caution regarding potential non-independence of observations.

## 3. Results

Table 2 shows that the baseline characteristics of the intervention and control groups were largely comparable. The majority of participants in both groups were male (50.9%). The mean age of participants in the intervention group was 13.86 years, whereas that of the control group was 13.93 years. Most participants reported never having had a romantic partner (42.1% in the intervention group and 64.9% in the control group, respectively). The majority of parents in both groups were living together (78.9%). The mean monthly allowance was 2438.60 Thai baht in the intervention group and 2345.61 Thai baht in the control group. Most participants spent their leisure time primarily using the internet (43.9% in the intervention group and 57.9% in the control group, respectively). In addition, most participants reported spending 3–6 h per day using the internet and social media (49.1% in the intervention group and 59.6% in the control group, respectively). Comparisons of baseline personal characteristics between the intervention and control groups indicated no statistically significant differences (Table 2).

### Sexual Health Literacy and Sexual Risk Behavior Prevention Results

The two-way mixed-design ANOVA revealed significant effects of group, time, and the group × time interaction on sexual health literacy scores (*p* < 0.001). In the intervention group, the mean sexual health literacy score increased from 70.86 (SD = 4.81) at the pre-test to 142.47 (SD = 3.43) at the post-test and slightly decreased but remained high at the one-month follow-up (127.88, SD = 4.85). In contrast, the control group showed almost no change across the three measurements, with mean scores of 71.33 (SD = 5.00), 71.28 (SD = 5.41), and 71.54 (SD = 7.53) at the pre-test, post-test, and one-month follow-up, respectively. The intervention demonstrated a very large effect size for sexual health literacy (η^2^ = 0.932) (Table 3).

Similarly, significant effects were found for sexual risk behavior prevention (*p* < 0.001). In the intervention group, the mean score increased from 55.04 (SD = 5.51) at the pre-test to 85.40 (SD = 2.35) at the post-test and remained high at the one-month follow-up (82.53, SD = 1.79). In contrast, the control group showed only minimal changes across the three measurements, with mean scores of 55.11 (SD = 4.74), 55.98 (SD = 6.03), and 54.88 (SD = 5.83) at the pre-test, post-test, and one-month follow-up, respectively. The intervention also demonstrated a large effect size for sexual risk behavior prevention (η^2^ = 0.791) (Table 3).

## 4. Discussion

The results of implementing the sexual health literacy promotion program (CoMMIT program) indicated significant improvements in sexual health literacy and preventive sexual health behaviors among students in the intervention group at both the post-intervention phase and the 1-month follow-up compared with baseline. These improvements were also significantly greater than those observed in the control group. These findings are consistent with previous studies demonstrating that school-based sexual health literacy interventions can effectively improve adolescents’ health related knowledge, decision-making skills, and behavioral intentions, particularly when active learning strategies are incorporated (Gómez-Lugo et al., 2022; Rodríguez-García et al., 2025; Estrada et al., 2024). Similarly, prior health literacy research suggests that improved literacy competencies are associated with more appropriate preventive health behaviors, supporting the conceptualization of sexual health literacy as a multidimensional determinant of adolescent preventive health behavior beyond simple knowledge acquisition.

Although the levels observed at the 1-month follow-up slightly decreased compared with the immediate post-intervention phase, they remained higher than baseline levels. Therefore, the findings should be interpreted as evidence of short-term maintenance rather than long-term sustainability of intervention effects. This pattern is consistent with evidence indicating that behavioral and attitudinal improvements from intensive educational interventions may gradually decline over time without continued reinforcement (Thongnopakun et al., 2021). Future studies should therefore investigate reinforcement strategies, such as booster sessions or extended program exposure, to support longer-term maintenance.

The CoMMIT program incorporated active and experiential learning strategies, including questioning, group discussion, reflection, hands-on activities, and scenario-based learning. These approaches are consistent with constructivist learning theory emphasizing active participation and contextualized knowledge construction (Doolittle et al., 2023). In addition, multimedia learning materials and realistic scenarios likely strengthened cognitive engagement, motivation, and decision-making processes.

Beyond the intervention content itself, contextual and procedural factors may also have contributed to the observed outcomes. The intervention group received structured learning sessions, multimedia-based educational materials, and closer interaction with facilitators throughout the five-week intervention period, which may have enhanced student engagement and learning processes. Potential Hawthorne effects may also have modestly contributed to the observed improvements. Future studies should therefore consider attention-matched control conditions to further isolate the specific effects of the intervention.

Importantly, the program emphasized the development of all six components of sexual health literacy: access to information, knowledge and understanding, communication skills, self-management, media literacy, and decision-making skills. These components were cultivated through a three-step active learning process (Ponrachom & Boonchuaythanasit, 2022), consisting of (1) stimulating thinking as an introduction to the lesson, (2) developing thinking through structured learning activities focused on preventing sexual risk behaviors, and (3) summarizing and reflecting on learning to consolidate knowledge gained from the instructional process. This multidimensional structure is consistent with contemporary health literacy frameworks emphasizing functional, interactive, and critical competencies and aligns with evidence suggesting that multidimensional and participatory approaches may better support adolescent behavioral development than single-domain approaches (Kabelka et al., 2025).

In contrast, the control group received routine classroom instruction at the secondary school level in accordance with the standard curriculum. This difference in pedagogical approach and engagement intensity may partly explain the between-group differences observed in this study and is consistent with previous research indicating that traditional sexuality education alone may not be sufficient to produce sustained improvements in behavioral competencies and decision-making capacities (Thongnopakun et al., 2021; Rodríguez-García et al., 2025). This instructional approach remained predominantly lecture-based, lacked integration with real-life contexts, and provided limited opportunities for learner participation and decision-making. Previous research suggests that conventional didactic sexuality education alone may not sufficiently improve adolescents’ behavioral competencies without participatory and skills-based components (Kabelka et al., 2025; Rodríguez-García et al., 2025). Evidence from recent research indicates that conventional school-based sexuality education focusing primarily on didactic instruction and risk avoidance does not consistently improve adolescents’ sexual behaviors or decision-making capacities without incorporating comprehensive, participatory, and skills-based components (Rodríguez-García et al., 2025).

As a result, sexual health literacy and preventive behaviors remained at insufficient levels, with no sustained improvement over time. Most participants reported having no romantic relationship or sexual experience at baseline. Therefore, improvements in sexual risk behavior prevention likely reflect increased preventive readiness, cognitive awareness, self-regulation, and self-protective intentions rather than direct behavioral change. Accordingly, the findings should be interpreted as reflecting preventive competencies and perceived behavioral capacity rather than actual observed sexual behavior, particularly during early adolescence, which is a developmental stage focused on building foundational decision-making competencies (World Health Organization, 2024a).

Given the sensitive nature of sexual health content among early adolescents, supportive and age-appropriate learning environments were maintained throughout the intervention to ensure psychological safety. Participation in discussions was voluntary, students were not required to disclose personal experiences, and no adverse reactions were reported during the study period.

Several methodological limitations should be considered. Although baseline characteristics were generally comparable between groups, a borderline difference in romantic relationship status (*p* = 0.078) may have influenced sexual health-related attitudes and decision-making. In addition, the quasi-experimental design and recruitment from different schools may introduce selection bias and school-level confounding factors, including differences in school environment, teaching practices, and peer norms. The relatively large effect sizes observed (η^2^ = 0.932 and 0.791) should also be interpreted cautiously, as they may partly reflect measurement-related characteristics, including potential floor and ceiling effects, reduced post-test variability, and alignment between intervention content and assessment constructs. Therefore, the magnitude of effects should be interpreted as context-specific and may not be directly generalizable to other settings or measurement instruments.

Overall, the findings suggest that the CoMMIT program represents a promising school-based approach for improving sexual health literacy and preventive competencies among early adolescents. However, the findings primarily support short-term effectiveness and preliminary maintenance rather than definitive long-term sustainability. Future research should employ randomized or cluster-randomized designs, include larger numbers of schools, extend follow-up periods, and utilize more externally validated measurement tools to strengthen causal inference and generalizability. Further research should also examine implementation across diverse cultural and educational contexts and identify which program components contribute most strongly to observed outcomes in order to optimize intervention design and scalability.

## 5. Conclusions

The CoMMIT program demonstrated significant effectiveness in improving sexual health literacy and preventive sexual risk behaviors among lower secondary school students. Participants in the intervention group showed consistently higher outcomes than the control group across post-intervention and 1-month follow-up assessments, indicating short-term effectiveness with partial maintenance over time.

These findings support the integration of multidimensional, active-learning-based sexual health literacy programs into school health education to strengthen adolescents’ cognitive and decision-making competencies during early adolescence.

However, the findings should be interpreted in light of several limitations, including the quasi-experimental design, limited number of schools, short follow-up period, potential school-level confounding, and possible measurement-related biases. In addition, the observed outcomes may reflect preventive intentions and perceived readiness rather than actual behavioral change, particularly because most participants reported no prior sexual experience. Differences in instructional intensity and participant engagement between groups may also have partially contributed to the observed effects.

Future research should employ randomized or cluster-randomized controlled designs, include larger and more diverse samples, extend follow-up duration, and use externally validated instruments to strengthen evidence on effectiveness and sustainability. Further studies should also examine the most impactful components of the program to support optimization and scalability in different educational and cultural contexts.

## Figures and Tables

**Table 1:** Summary of the sexual health literacy promotion program (CoMMIT program).

No.	Activity Title	Content	Activities
1	Co: Comprehend after Access	- Guidelines for the prevention of sexual risk behaviors - Approaches to searching for information on the internet - Methods for distinguishing information obtained from online media	- Video-based media presentation - Group relationship-building activities and brainstorming - Questioning techniques - Practical exercises (brainstorming/matching) Focus: Strengthening components of sexual health literacy, including access to information and knowledge and understanding
2	M: Managing and Communication Oneself	- Effective communication styles - Steps for goal setting and strategies for achieving set goals	- Self-directed learning and presentation using mind maps - Online media - Knowledge sharing and exchange Focus: Strengthening components of sexual health literacy, including communication skills and self-management
3	M: Media Literacy for Decision	- Methods for identifying real and fake news in online environments - Techniques for verifying the accuracy and credibility of information—Approaches to analyzing options for refusing or avoiding sexual risk behaviors	- Observation through role models - Practical exercises and knowledge exchange Focus: Strengthening components of sexual health literacy, including media literacy and decision-making skills
4	I: I Can Do It	- Guidelines and methods for preventing sexual risk behaviors: (1) refraining from substance and alcohol use (2) avoiding sexually arousing media (3) avoiding intimate physical contact in private settings	- Questioning techniques - Viewing video-based media - Game-based activities - Practical exercises and knowledge exchange Focus: Strengthening components of sexual health literacy, including access to information, knowledge and understanding, communication skills, self-management, media literacy, and decision-making skills
5	T: Training SHL for Oneself and Others	- Summary and knowledge exchange on guidelines and methods for preventing sexual risk behaviors	- Practical exercises - Brainstorming - Group discussion Focus: Strengthening components of sexual health literacy, including access to information, knowledge and understanding, communication skills, self-management, media literacy, and decision-making skills

**Table 2:** Baseline characteristics of study participants.

Characteristics	Intervention Group (*n* = 57)		Control Group (*n* = 57)		*p*-Value
	*n*	%	*n*	%	
Sex					
Male	29	50.9	29	50.9	1.000 ^a^
Female	24	42.1	24	42.1	
LGBTQ+	4	7.0	4	7.0	
Age (years), Mean ± SD	13.86 ± (0.350)		13.93 ± (0.258)		0.226 ^b^
Romantic relationship status					
Currently in a relationship	9	15.8	8	14.0	0.078 ^a^
Previously, but not currently	14	24.6	7	12.3	
In a dating stage	10	17.5	5	8.8	
Never had a relationship	24	42.1	37	64.9	
Parental marital status					
Living together	45	78.9	45	78.9	0.968 ^a^
Separated/Divorced	9	15.8	8	14.0	
One parent deceased	3	5.3	4	7.1	
Monthly allowance (THB), Mean ± SD	2438.60 ± (642.471)		2345.61 ± (551.320)		0.409 ^b^
Primary leisure activity					
Internet use	25	43.9	33	57.9	0.536 ^a^
Watching television at home	14	24.6	8	14.0	
Going out	9	15.8	8	14.0	
Reading	7	12.3	7	12.3	
Part-time work	2	3.4	1	1.8	
Frequency of internet and social media use					
<3 h/day	17	29.8	15	26.4	0.471 ^a^
3–6 h/day	28	49.1	34	59.6	
>6 h/day	12	21.1	8	14.0	

Notes: ^a^ Chi-Square test, ^b^ Independent *t*-test.

**Table 3:** Sexual health literacy and sexual risk behavior prevention mean scores and two-way mixed-design ANOVA results.

Scales and Measurements	Intervention Group (*n* = 57)	Control Group (*n* = 57)		Group	Time	Group * Time Interaction
	Mean (SD)	Mean (SD)				
Sexual Health Literacy						
Pre-test	70.86 (4.81) ^A,a^	71.33 (5.00) ^A,a^	F	4865.092	1533.210	1530.078
Post-test	142.47 (3.43) ^A,b^	71.28 (5.41) ^B,a^	*p*	<0.001	<0.001	<0.001
Follow-up (1 month)	127.88 (4.85) ^A,b^	71.54 (7.53) ^B,a^	η^2^	0.977	0.932	0.932
Sexual Risk Behavior Prevention						
Pre-test	55.04 (5.51) ^A,a^	55.11 (4.74) ^A,a^	F	448.355	448.355	424.017
Post-test	85.40 (2.35) ^A,b^	55.98 (6.03) ^B,a^	*p*	<0.001	<0.001	<0.001
Follow-up (1 month)	82.53 (1.79) ^A,b^	54.88 (5.83) ^B,a^	η^2^	0.904	0.800	0.791

F = two-way mixed-design ANOVA; η^2^ = partial eta squared. Different uppercase letters in the same row indicate significant differences between intervention and comparison groups. Different lowercase letters in the same column indicate significant differences between measurement times.

## Data Availability

The original contributions presented in this study are included in this article. Further inquiries can be directed to the corresponding author.
